# Galectin-14 promotes hepatocellular carcinoma tumor growth *via* enhancing heparan sulfate proteoglycan modification

**DOI:** 10.7555/JBR.37.20230085

**Published:** 2023-11-15

**Authors:** Liming Gou, Gang Yang, Sujuan Ma, Tong Ding, Luan Sun, Fang Liu, Jin Huang, Wei Gao

**Affiliations:** 1 Jiangsu Key Lab of Cancer Biomarkers, Prevention and Treatment, Collaborative Innovation Center for Personalized Cancer Medicine, Key Laboratory of Human Functional Genomics of Jiangsu Province, National Health Commission Key Laboratory of Antibody Techniques, School of Basic Medical Sciences, Nanjing Medical University, Nanjing, Jiangsu 211166, China; 2 Core Laboratory, the Affiliated Sir Run Run Hospital of Nanjing Medical University, Nanjing, Jiangsu 211166, China; 3 Department of Gastroenterology, the Affiliated Changzhou No.2 People's Hospital of Nanjing Medical University, Changzhou Medical Center of Nanjing Medical University, Changzhou, Jiangsu 213000, China

**Keywords:** hepatocellular carcinoma, galectin-14, heparan sulfate proteoglycans, co-receptor

## Abstract

Hepatocellular carcinoma (HCC) is a highly heterogeneous malignancy and lacks effective treatment. Bulk-sequencing of different gene transcripts by comparing HCC tissues and adjacent normal tissues provides some clues for investigating the mechanisms or identifying potential targets for tumor progression. However, genes that are exclusively expressed in a subpopulation of HCC may not be enriched or detected through such a screening. In the current study, we performed a single cell-clone-based screening and identified galectin-14 as an essential molecule in the regulation of tumor growth. The aberrant expression of galectin-14 was significantly associated with a poor overall survival of liver cancer patients with database analysis. Knocking down galectin-14 inhibited the proliferation of tumor growth, whereas overexpressing galectin-14 promoted tumor growth *in vivo*. Non-targeted metabolomics analysis indicated that knocking down galectin-14 decreased glycometabolism; specifically that glycoside synthesis was significantly changed. Further study found that galectin-14 promoted the expression of cell surface heparan sulfate proteoglycans (HSPGs) that functioned as co-receptors, thereby increasing the responsiveness of HCC cells to growth factors, such as epidermal growth factor and transforming growth factor-alpha. In conclusion, the current study identifies a novel HCC-specific molecule galectin-14, which increases the expression of cell surface HSPGs and the uptake of growth factors to promote HCC cell proliferation.

## Introduction

Primary liver cancer is the sixth most frequently diagnosed and ranks the third-leading cause of cancer-related death, with an estimated 906000 new cases and 830000 deaths worldwide ^[ 1] ^. Hepatocellular carcinoma (HCC) is the most common type of primary liver cancer, accounting for approximately 90% of cases. Viral hepatitis, alcohol, and non-alcoholic fatty liver disease are the main risk factors for HCC development ^[ 2] ^. The prognosis of HCC patients remains poor, and the tumor recurrence rate five years after resection is more than 70% ^[ 3] ^. The targeted therapy has shown significant advantages in the treatment of advanced HCC. The Food and Drug Administration has approved several drugs, such as Sorafenib, Lenvatinib, Cabozantinib, and other immune checkpoint inhibitors, which have improved the survival rate of patients with advanced HCC ^[ 4– 5] ^. However, there are still many limitations in the targeted therapy of HCC at present ^[ 6] ^. Hence, it is important to explore the underlying mechanisms responsible for HCC tumorigenesis to identify promising targets for HCC therapy.

HCC is a highly heterogeneous malignancy and lacks an effective treatment ^[ 7] ^. Bulk-sequencing of different gene transcripts by comparing HCC tissues and adjacent normal tissues provides some clues for investigating the mechanisms or identifying potential targets for tumor progression. However, genes that are exclusively expressed in a subpopulation of HCC may not be enriched or detected through such a screening. Therefore, comparing single tumor cell clones exhibiting different phenotypes may provide us with more detailed information that represents the heterogeneity of HCC ^[ 8– 10] ^.

By doing this, we found that galectin-14 might be a potential molecule associated with HCC tumor growth. Galectin-14 is a member of the galectin family. Galectins have a high specific affinity with β-galactosides by their conserved carbohydrate recognition domain ^[ 11] ^. The activity can be inhibited by lactose, galactose, or related carbohydrates, such as *N*-acetylgalactosamine. To date, 19 mammalian galectins have been identified. According to the number and structure of carbohydrate recognition domains, galectins can be divided into three types: prototype, tandem-repeat type, and chimeric type ^[ 12] ^. They are involved in many cellular functions, including development, inflammation, metabolism, pre-mRNA splicing, immune cell activity regulation, and microbial recognition in the innate immune system ^[ 13– 14] ^.

To date, many galectins are either aberrantly expressed or dysfunctional in hepatocytes, including galectin-1, galectin-3, galectin-4, and galectin-9 ^[ 15] ^. Galectin-1 and galectin-3 expression levels are aberrantly upregulated in HCC, significantly increasing the migration and invasion abilities of HCC cells ^[ 16– 18] ^. Galectin-4 expression is downregulated in HCC, and its low expression is closely associated with a poor prognosis of HCC ^[ 15] ^. Galectin-9 is specifically expressed in certain hepatitis B virus-associated HCC, and it is significantly associated with a poor prognosis ^[ 19] ^. It has been reported that specific expression of galectin-9 leads to immunosuppressive features in hepatitis B virus-associated HCC ^[ 20] ^. All of these findings indicate that galectins play a role in HCC progression through multiple mechanisms.

Heparan sulfate proteoglycans (HSPGs) are a type of glycosaminoglycan that assembles sulfated sugar residues in the format of a copolymer consisting of GlcNAcα4GlcAβ4 linked to serine residues in core proteins through xylose-linkage ^[ 21] ^. HSPGs are mainly located on the cell surface, such as hepatocytes and leukocytes. As part of extracellular matrix (ECM), HSPGs function as the co-receptor of many essential signaling pathways by concentrating growth factors on cell surface, therefore participating in both physiological and pathological processes ^[ 22– 24] ^. For example, fibroblast growth factor 10 (FGF10) binds to HSPGs and FGF receptor (FGFR) to induce the recruitment of protein adaptors to FGFR and signaling activation, resulting in cell proliferation, differentiation, and invasion ^[ 25] ^. It has been reported that there are four known HSPGs and three lectins (Mind the Gap, Lectin-GalC1, and galectin) working in the synaptomatrix to control multiple *trans*-synaptic signaling pathways ^[ 26] ^. However, the correlation between the expression levels of HSPGs and galectins in tumors, especially in HCC, is still unknown.

In the current study, we identified galectin-14 as a novel molecule specifically expressed in HCC *via* a single cell-clone-based screening. The aberrant expression of galectin-14 was associated with a poor prognosis of HCC. Galectin-14 promoted HCC cell proliferation by increasing the expression level of cell surface HSPGs to sensitize tumor cells responding to growth factors.

## Materials and methods

### Cells and reagents

Hepatocellular carcinoma cell lines, *i.e.*, Huh-7, LO2, LM3, JHH-7, MHCC-97H, SNM-398, Hep3B and HepG2, ovarian cancer cell line (OVCAR3), and human embryonic kidney cell line (HEK-293T) were purchased from the American Type Culture Collection (Manassas, VA, USA). Huh-7 sc1 and sc22 cells were screened from Huh-7 cells *via* single cell clone screening. Cells were maintained at 37 ℃, 5% CO _2_, in DMEM (HyClone, South Logan, UT, USA) supplemented with penicillin (100 U/mL), streptomycin (100 μg/mL; HyClone), and 10% fetal bovine serum (VACCA, St. Louis, MO, USA). 2-DG (5 mmol/L; Sigma, St. Louis, MO, USA) was used to treat wild type and galectin-14 knockdown Huh-7 cells for 24 h before detecting the expression of heparan sulfate on the cell surface. Epidermal growth factor (EGF; 5 ng/mL; Genscript, Nanjing, Jiangsu, China) and transforming growth factor alpha (TGF-α; 5 ng/mL; Genscript) were used to treat wild type and galectin-14 knockdown Huh-7 cells before cell proliferation analysis.

### Patient samples

The HCC tumor tissue specimens used in the current study were obtained from patients recruited from Jiangsu Taizhou People's Hospital (Taizhou, Jiangsu, China) between November 2022 and March 2023. The current study was approved by the Ethical Committee of the participating hospital (Approval No. KY-2022-172-01), and all patients provided a written informed consent. The procedure for the current study conformed to the provisions of the Declaration of Helsinki.

Patients with confirmed diagnoses of HCC, based on tests for alpha-fetoprotein and des-gamma-carboxy prothrombin, were included. Patients with human immunodeficiency virus infection or other causes of hepatocellular injury were excluded. None of the patients received anti-tumor therapies before diagnosis.

### 3 D tumor spheroid culture

Huh-7 and Huh-7 single clone cells at the logarithmic growth stage were seeded with 100 cells/well in U-type ultra-low adsorption 96-well cell culture plate (Corning, New York, USA) with 10 replicate wells. Cell culture plates were taken out every 48 h, and cell spheroid formation and proliferation were observed and photographed.

### Lentiviral infection of tumor cells

Lentivirus-mediated overexpression of galectin-14 was performed in Huh-7 and Huh-7 sc1 cells by galectin-14-expression vector. Lentivirus-mediated konckdown of galectin-14 was performed in Huh-7 sc22 cells by small hairpin RNA targeting *LGALS14*, the gene encoding galectin-14. The plasmid pLVX (10 μg; Plasmid #P0961, MiaoLingBio, Wuhan, China) carrying the full-length *LGALS14* or plasmid pGreenPuro (10 μg; Plasmid #P0420, MiaoLingBio) carrying *LGALS14* shRNA (5′-GATCCGCATCTATGTGCGTCACAAGGCTTCCTGTCAGACCTTGTGACGCACATAGATGCTTTTTG-3′) was co-transfected with plasmids psPAX2 (7.5 μg; Plasmid #12260, Addgene, Watertown, MA, USA) and pMD2G (5 μg; Plasmid #12259, Addgene) into HEK-293T cells in 100 mm cell-culture dishes (Jet Biofil, Guangzhou, China). After 48 and 72 h, virus-containing supernatants were collected and filtered through a 0.45 mm PES Syringe Filter (Thermo Fisher Scientific, Waltham, MA, USA), and then used to infect tumor cells in the presence of 10 mg/mL polybrene (Yeasen, Shanghai, China). Finally, puromycin (3 μg/mL; Yeasen) was added to screen positive infected Huh-7, Huh-7 sc1, Huh-7 sc22, and OVCAR3 cells for five days. Stable cell lines were maintained in puromycin (1 μg/mL) containing medium.

### Western blotting

The cells were lysed in a lysis buffer (Beyotime, Shanghai, China) containing protease inhibitor (Beyotime) and PMSF (Beyotime). The total protein was then separated by 15% SDS-PAGE (80 V, 30 min for concentrated gel, and 120 V, 80 min for separation gel) and transferred to PVDF film (pore diameter 0.22 μm). The membrane was blocked in 5% skim milk for 1 h and then incubated with rabbit anti-galectin-14 (1∶1000 dilution; Cat. #ab150427, Abcam, Cambridge, MA, USA) overnight at 4 ℃. After washing with Tris-buffered saline with Tween 20 (TBST), the membrane was incubated with HRP-conjugated goat anti-rabbit secondary antibody (1∶1000 dilution; Cat. #111-035-003, Jackson, West Grove, PA, USA) for 1 h. After the film was washed four times, the protein bands were exposed with an enhanced chemiluminescence (Tanon, Shanghai, China), and the grayscale analysis was performed with Image J software.

### RNA extraction and quantitative RT-PCR (qRT-PCR)

Total RNA was isolated from either tissues or cells using TRIzol reagent (Beyotime) following the manufacturer's protocol. Then, 1 μg of total RNA was transcribed into cDNA using a reverse transcriptase kit (Vazyme, Nanjing, China). Each qRT-PCR procedure was repeated thrice in 20 μL solution containing the SYBR Green real-time PCR Master Mix (Vazyme). Primer sequences are as follows: galectin-14 forward, 5′-CTTTTGTCAAGGACCCACAGC-3′; galectin-14 reverse, 5′-AAGCACTCTGGTCAGGGAGA-3′; α-tubulin forward, 5′-CCAAGCTGGAGTTCTC-3′; and α-tubulin reverse, 5′-CAATCAGAGTGCTCCAG-3′.

### TissueScan cDNA array

The human normal tissue cDNA array (Cat. #HMRT104, OriGene, Rockville, MD, USA) was removed from the −20 ℃ refrigerator, centrifuged once for a short time, and equilibrated to room temperature. Then, the self-sealing bag was opened, and the 96-well plate of the cDNA array was removed. The galectin-14 was tested according to the instructions provided.

### Animal tests

BALB/c nude mice were purchased from the Model Animal Research Center of Nanjing University. All mice were fed with standard specific pathogen-free conditions at the Experimental Animal Center of Nanjing Medical University. A total of 5 × 10 ^6^ Huh-7, Huh-7 sc22 and Huh-7 sc1 cells were suspended in 200 µL of PBS and subcutaneously inoculated into the right flank of 4- to 6-week-old female mice that were shaved with an electric clipper. Tumor size and weight were measured regularly with a digital caliper. Tumor volume was calculated as length × width ^2^ × 0.5. Mice were euthanized when the tumor size reached 2000 mm ^3^. All animal experiments were approved by the Animal Ethical and Welfare Committee of Nanjing Medical University (Approval No. IACUC-2104034). All procedures were performed according to institutional guidelines for the care and use of animals.

### RNA-seq analysis

Subcutaneous tumor tissues obtained from the mice inoculated with Huh-7, Huh-7 sc22, or Huh-7 galectin-14 knockdown cells were stored in 1 mL of TRIzol ( *n* = 3 for each group). RNA was then extracted and subjected to pre-sequencing quality control (A260/A280 at around 2.0). The library and sequencing were completed by Shanghai Yaoming Kangde Chemical Technology Company (Shanghai, China). The library was built on Illumina Hiseq, with 150 bp sequencing length and two-terminal sequencing. The sequencing data were aligned to the human genome sequence (version HG19) by STAR (v2.5.1b) software. Transcript abundance was quantified as fragments per kilo base of exon per million fragments mapped. Subsequently, differential expression was assessed using DEseq2 (1.22.2). The threshold for differential gene screening was set to false discovery rate < 0.05 and |log _2_(fold change)| ≥ 1.

### Cell counting kit-8 (CCK-8) assay

Cell proliferation was determined by the CCK-8 assay using the CCK-8 reagents (Vazyme). Cells were seeded at a density of 3000 cells/well in 96-well plates. After being cultured for the indicated time, 10 µL of CCK8 solutions were added and incubated for 1 h at 37 ℃. The absorbance values were measured at 450 nm.

### Flow cytometry

A single-cell suspension was incubated with 5 µg/mL of the indicated antibodies for 1 h on ice, and then incubated with a 1∶200 dilution of goat anti human IgG-APC (Cat. #409306, BioLegend, San Diego, CA, USA) for 1 h on ice. The cells were analyzed using FACS Calibur (BD Biosciences, San Jose, CA, USA).

### Non-targeted metabolomics analysis

Wild-type and galectin-14 knockdown Huh-7 cells (2 × 10 ^7^ for each) were trypsin digested and collected. After washing with PBS, each group of cells was equally divided into four aliquots, with 5 × 10 ^6^ cells per aliquot. The cells were snap frozen in liquid nitrogen for 15 min and then stored at −80 ℃ until delivery. The non-targeted metabolomics assays were performed by Shenzhen Huada Gene Technology Company (Shenzhen, China). Non-targeted metabolomics analysis was performed based on liquid chromatography with tandem mass spectrometry (LC-MS/MS) technique using a high resolution mass spectrometer Q Exactive (Thermo Fisher Scientific). Data were acquired in both positive and negative ion modes separately to improve metabolite coverage. Compound Discoverer 3.1 software (Thermo Fisher Scientific) was used for the LC-MS/MS data processing, including peak extraction, peak alignment, and compound identification. The UW self-developed metabolomics R package metaX and metabolome information analysis process were used for data pre-processing, statistical analysis, metabolite classification, and functional annotation. The raw multivariate data were downscaled by principal component analysis to analyze groupings, trends (similarities and differences within and between sample groups), and outliers (presence of outlier samples) of the observed variables in the dataset. The variable important in projection (VIP) values of the first two principal components of the partial least squares-discriminant analysis (PLS-DA) model were used, combined with the fold change and Student's *t*-test obtained from univariate analysis to screen the differential metabolites. Finally, significantly differentiated metabolites were required to meet the following criteria: VIP ≥ 1, fold-change ≥ 1.2 or ≤ 0.83 and *Q*-value < 0.05 for the first two principal components of the PLS-DA model.

### siRNA transfection

The expression of EXT1 was silenced using siRNA targeting *EXT1* (UGAGAGACUGCCGCCAAGUAA, Genepharma, Shanghai, China). When cells were confluent to approximately 50%, the culture medium was replaced with fresh medium. The siRNA and siRNA-mate (Genepharma) were added to opti-MEM and incubated for 10 min, and then added to the cells for 48 h. Cell identification and subsequent experiments were then performed.

### Statistical analysis

All *in vitro* experimental data were presented as mean ± standard deviation of at least three replicates. The experimental data of subcutaneous transplanted tumors in nude mice were presented as mean ± standard error of the mean. All statistical analyses were conducted using GraphPad Prism 8.0 (GraphPad Software Inc., San Diego, CA, USA). Two-tailed Student's *t*-test of the means and two-way ANOVA were used for statistical analysis, with *P* < 0.05 considered statistically significant.

## Results

### Single-cell clone-based screening to identify the key regulators related to HCC tumor growth

**Figure 1:**
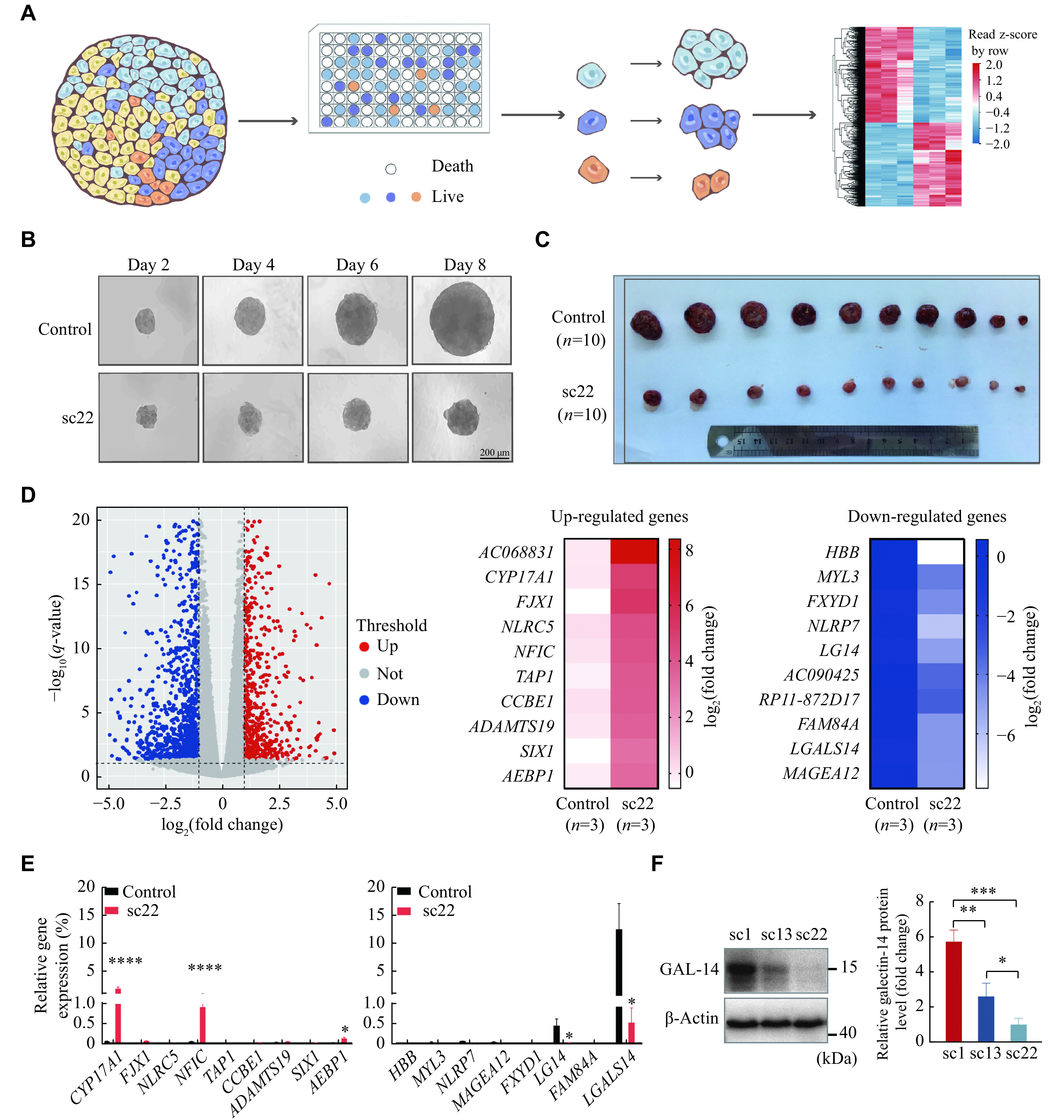
Galectin-14 was to be downregulated by single cell clone-based RNA-seq screening.

The heterogeneity is one of the key malignant features of tumor cells and is a major obstacle hampering the target discovery for cancer therapy ^[ 27] ^. Here we established a strategy of phenotype-based single cell cloning screening: single tumor cells were expanded in culture and then subjected to RNA-seq analysis based on their proliferation differences to obtain functional genes expressed in tumor cell subsets ( ***Fig. 1A***). We seeded 500 Huh-7 cells as single cell culture and obtained 22 single cell clones. We named these single cell clones according to their growth rate, as the single cell clone 1 (sc1) showed the fastest growth rate, single cell clone 13 (sc13) showed the moderate growth rate, and single cell clone 22 (sc22) showed the slowest growth rate. We performed *in vitro* and *in vivo* experiments and confirmed the attenuated growth of Huh-7 sc22 cells, compared with the original Huh-7 cells ( ***Fig. 1B*** and ***1C***). We obtained the subcutaneous tumor tissues from the mice inoculated with Huh-7 and Huh-7 sc22 cells to perform RNA sequencing analysis, and identified a panel of differentially expressed genes between the two groups ( ***Fig. 1D***). We next verified the expression of these candidate genes by qRT-PCR. The results showed that *LGALS14*, the gene encoding galectin-14, was the one with the most significant expression change between the Huh-7 and Huh-7 sc22 cells ( ***Fig. 1E***). We further confirmed the expression of galectin-14 in different monoclonal cells derived from Huh-7 cells, and found that the fastest-growing Huh-7 sc1 cells had a highest expression level of galectin-14, the medium-growing Huh-7 sc13 cells had a moderate expression level of galectin-14, and no expression of galectin-14 was detected in the slowest-growing Huh-7 sc22 cells ( ***Fig. 1F***).

### Galectin-14 aberrantly expressed in HCC tissues and associated with a poor survival of pan-cancer

**Figure 2:**
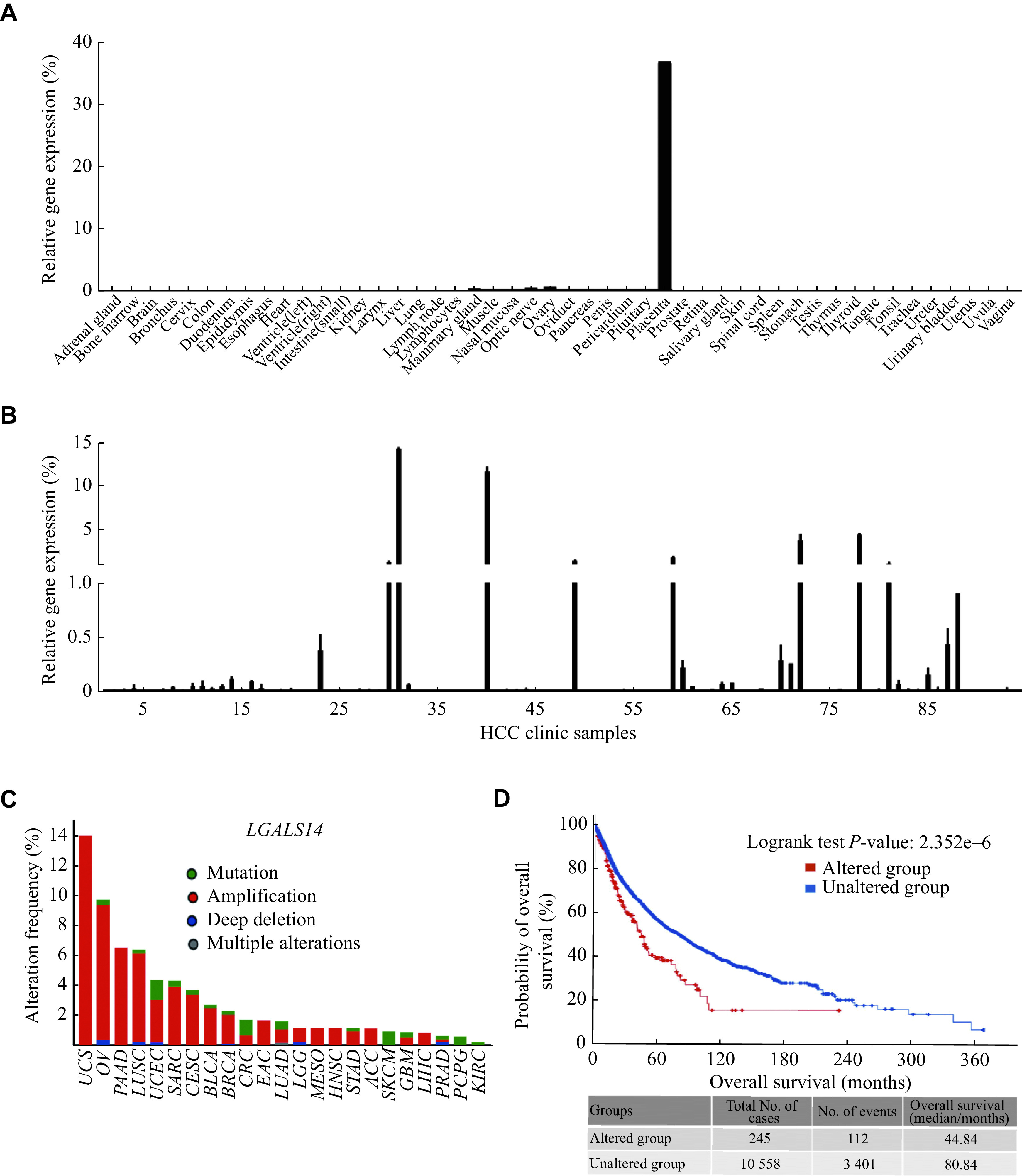
Clinical relevance analysis of galectin-14 and tumors.

To valid the clinical relevance of galectin-14, we first performed a normal tissue cDNA array and found that galectin-14 expressed only in normal human placenta ( ***Fig. 2A***). However, we found that 20% of our collected HCC tumor tissue samples expressed galectin-14 ( ***Fig. 2B***). Moreover, by analyzing The Cancer Genome Atlas (TCGA) program database, we found that besides HCC, galectin-14 aberrantly expressed in a series of tumor types, especially induced by gene copy number amplification ( ***Fig. 2C***), and aberrant alterations of the galectin-14 gene in the genome showed a significant association with a poor survival in patients with pan-cancer ( ***Fig. 2D***). Altogether, these results suggest that galectin-14 may play important roles in tumor progression and has the potential to be a therapeutic target for HCC.

### Galectin-14 promoted Huh-7 cell proliferation and xenograft tumor growth

**Figure 3:**
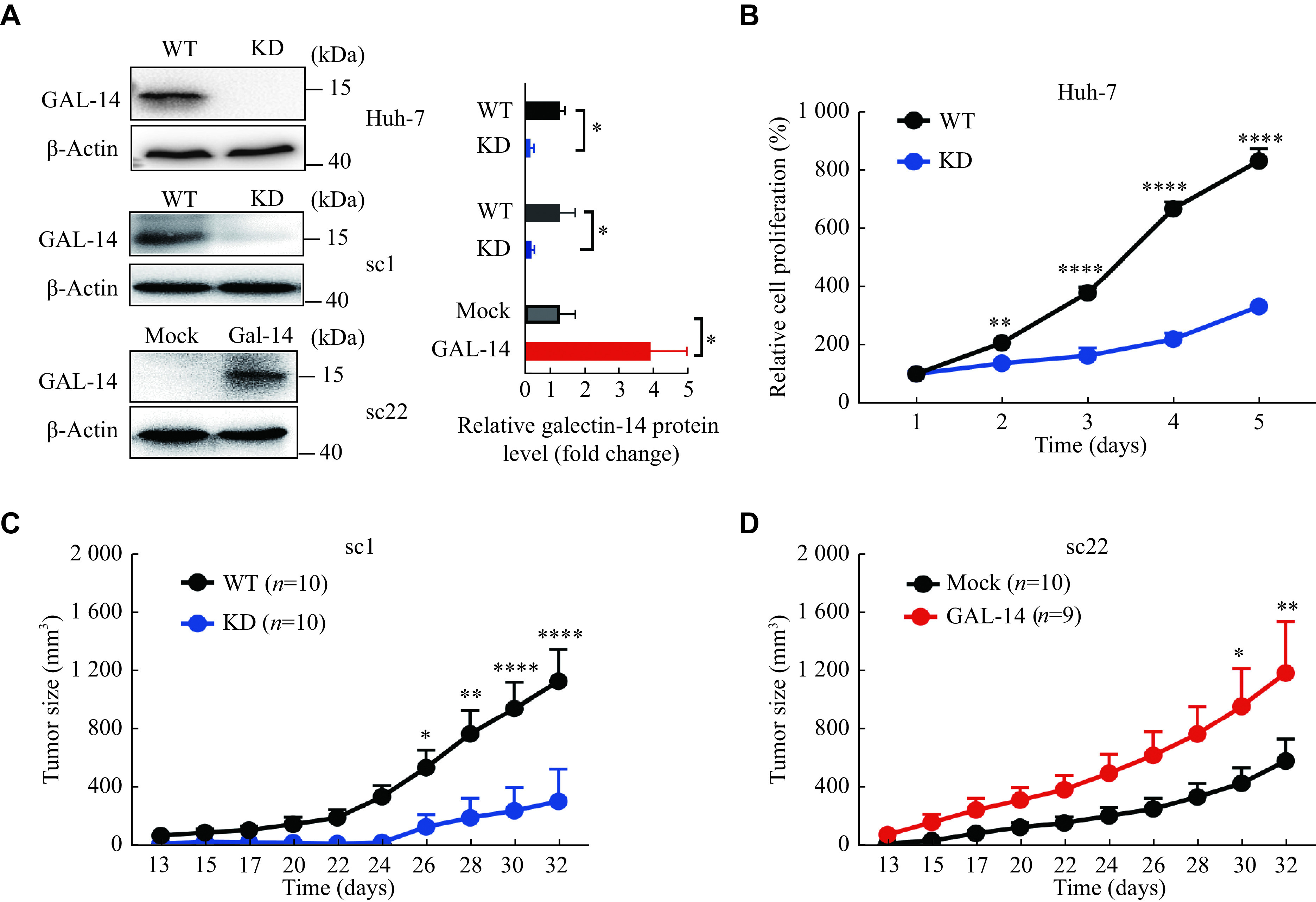
Galectin-14 promoted the proliferation of tumor cells *in vitro* and *in vivo*.

We detected galectin-14 expression in several HCC cell lines and found that galectin-14 was only expressed in Huh-7 cells ( ***Supplementary Fig. 1 ***, available online). To investigate the function of galectin-14 in HCC, we knocked down galectin-14 in Huh-7 and Huh-7 sc1 (galectin-14 positive) cells, and overexpressed galectin-14 in sc22 (galectin-14 negative) cells ( ***Fig. 3A***). *In vitro* proliferation assays showed that galectin-14 knockdown significantly inhibited proliferation of Huh-7 cells ( ***Fig. 3B***). We also observed a similar phenomenon in OVCAR3, a galectin-14 positive-expressing ovarian cancer cell line ( ***Supplementary Fig. 2A*** and ***2B ***, available online). These data indicate probable role of galectin-14 in regulating tumor growth. We further investigated the tumor-promoting role of galectin-14 by xenograft experiments *in vivo*, and found that knocking down galectin-14 in Huh-7 sc1 cells significantly inhibited tumor growth, while overexpressing galectin-14 in Huh-7 sc22 cells promoted tumor growth ( ***Fig. 3C*** and ***3D***). These observations indicate that galectin-14 can be an important regulatory molecule promoting HCC tumor growth.

### Galectin-14 affected the expression of HSPGs in Huh-7 cells

**Figure 4:**
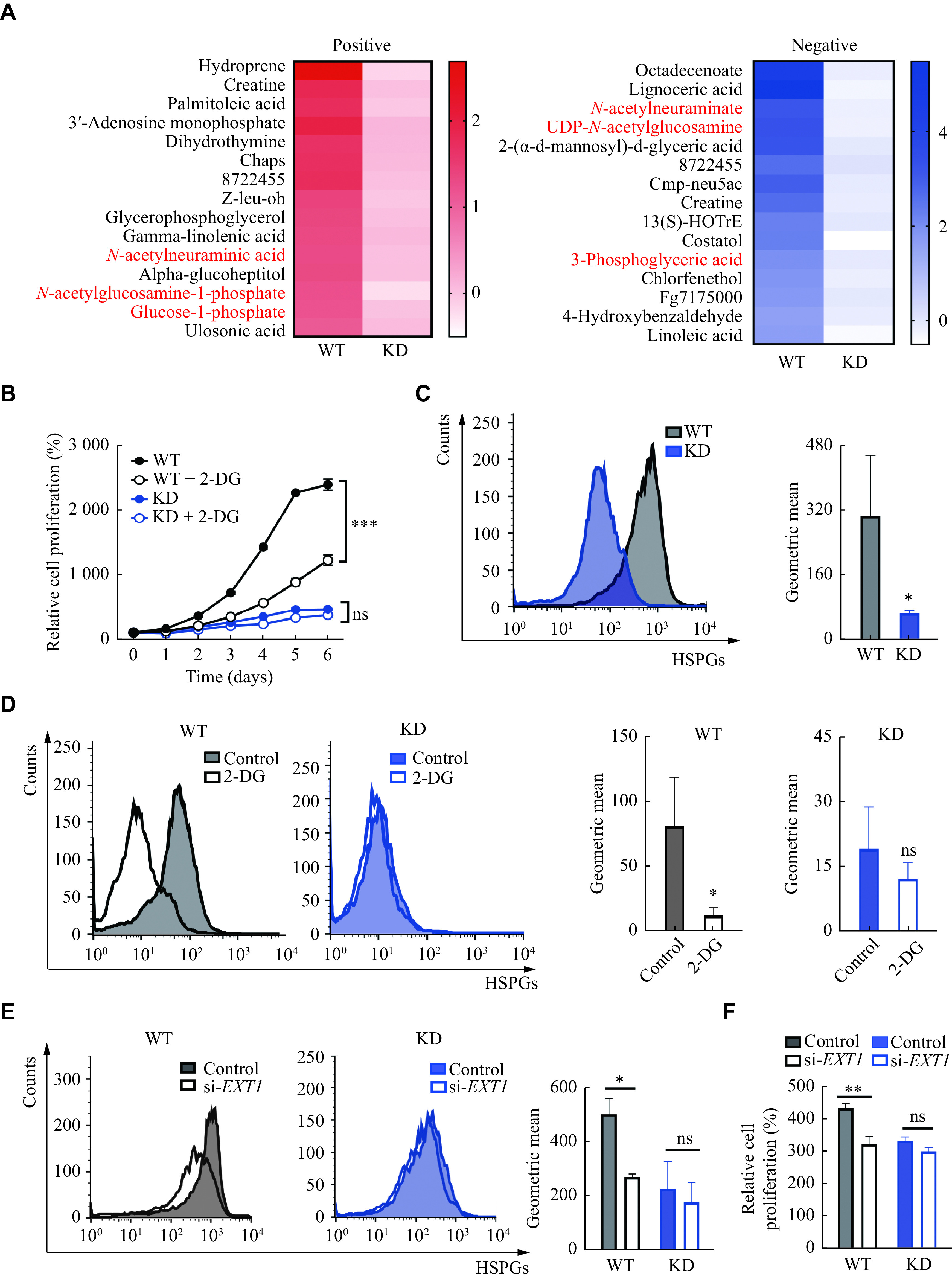
Galectin-14 affected the expression of HSPGs in HCC.

To investigate the mechanisms of galectin-14 in regulating HCC cell proliferation, we first detected whether the function of galectin-14 depended on glycolytic metabolism or not. We performed an untargeted metabolomics analysis in wild-type and galectin-14 knockdown Huh-7 cells, and analyzed the combination of the top 15 metabolites with the most significant changes in content in positive and negative modes. The results showed that the content of metabolic intermediates correlated with the glucose metabolism. Especially, those of the glycoside synthesis pathway was significantly changed in the galectin-14 knockdown Huh-7 cells, compared with the wild-type Huh-7 cells, including the intermediate metabolite of glycolytic pathway (3-phosphoglyceric acid) and the key intermediate metabolites of glucosamine synthesis pathway (glucose-1-phosphate, *N*-acetylglucosamine-1-phosphate, and UDP- *N*-acetylglucosamine) ( ***Fig. 4A***). These results indicate that galectin-14 is involved in the regulation of glycolytic metabolism. We then treated wild-type and galectin-14 knockdown Huh-7 cells with 2-DG, a glucose analog that can inhibit the glucose metabolic process ^[ 28] ^, and detected cell proliferation. The results showed that the proliferation of wild-type Huh-7 cells was significantly inhibited by 2-DG, whereas the proliferation of galectin-14 knockdown Huh-7 cells was much less affected ( ***Fig. 4B***). These results suggest that galectin-14 may promote HCC cell proliferation by mediating the glycolytic metabolism. Therefore, we finally compared the glycosylation patterns of wild-type and galectin-14 knockdown Huh-7 cells, and found that HSPG expression was significantly reduced when galectin-14 was knocked down in Huh-7 cells ( ***Fig. 4C***). In addition, we found that knocking down galectin-14 in OVCAR3 cells also reduced the expression of HSPGs, which was consistent with what we observed in Huh-7 cells ( ***Supplementary Fig. 2C ***, available online). These data indicate general role of galectin-14 in regulating HSPGs synthesis.

Further, we detected the expression of HSPGs in 2-DG treated wild type and galectin-14 knockdown Huh-7 cells, and found that 2-DG treatment significantly inhibited the expression of HSPGs in wild type Huh-7 cells, but did not affect the expression of HSPGs in galectin-14 knockdown Huh-7 cells ( ***Fig. 4D***). These results indicate that the expression of HSPGs is mediated by galectin-14. Because EXT1 functions as a key enzyme involved in the heparan sulfate chain extension step, we interfered the expression of EXT1 using siRNA in wild type and galectin-14 knockdown Huh-7 cells ( ***Supplementary Fig. 3 ***, available online) ^[ 29] ^. The HSPG expression on cell surface as well as the cell proliferation were subsequently detected. The results showed that the inhibition of EXT1 expression by RNA interference led to a signicantly decrease of HSPG expression in wild type Huh-7 cells, but had no significant effect on HSPG expression in galectin-14 knockdown Huh-7 cells ( ***Fig. 4E***). Meanwhile, the proliferation of wild-type Huh-7 cells was significantly inhibited when EXT1 was knocked down, whereas the proliferation of galectin-14 knockdown Huh-7 cells was not significantly affected ( ***Fig. 4F***). These results suggest that galectin-14 promotes HCC cell proliferation by mediating the expression of cell surface HSPGs, whose synthesis is limited by EXT1.

### Galectin-14 sensitized Huh-7 cells to growth factor treatment

**Figure 5:**
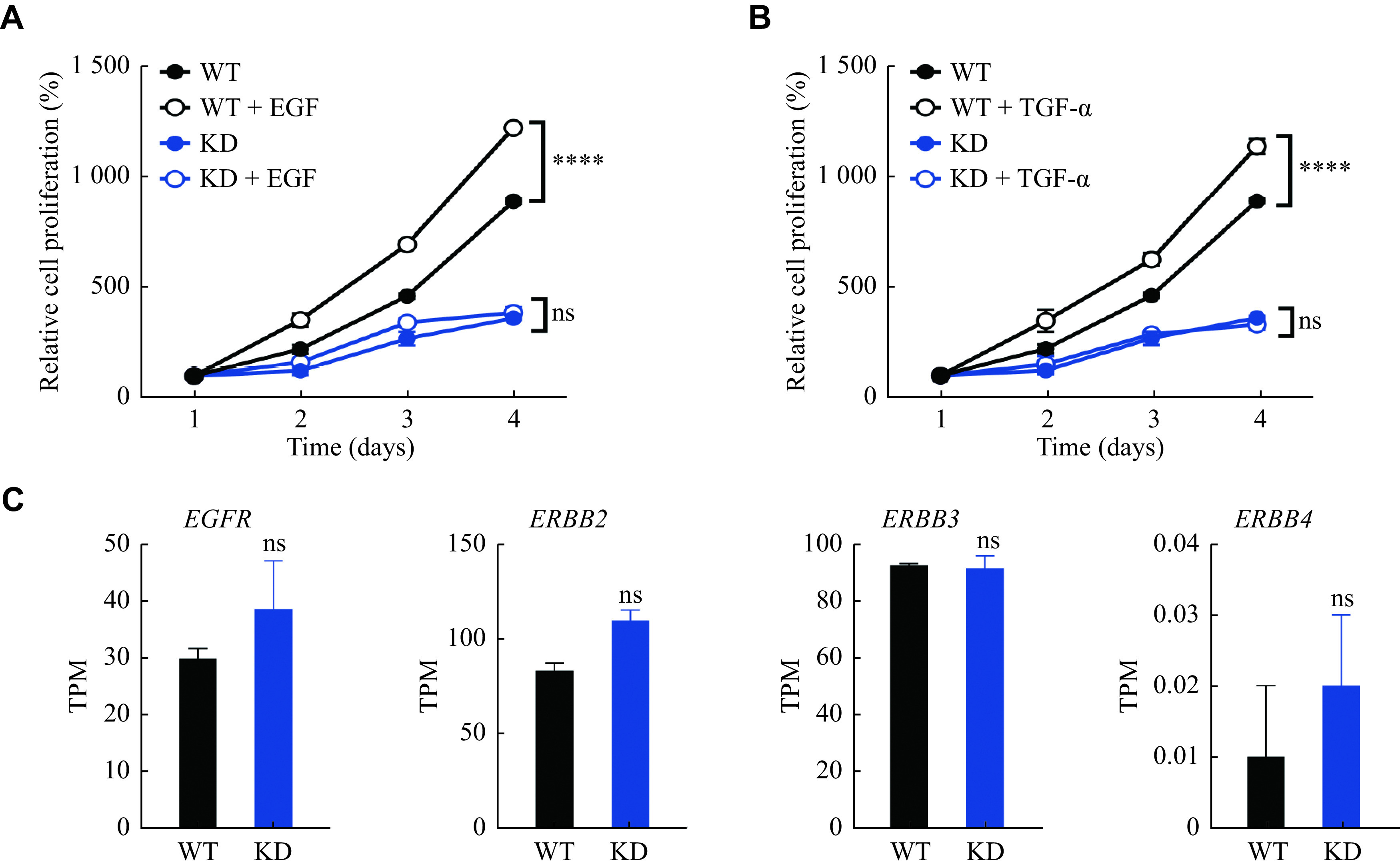
Galectin-14 sensitized the tumor cells responding to growth factor treatment.

**Figure 6:**
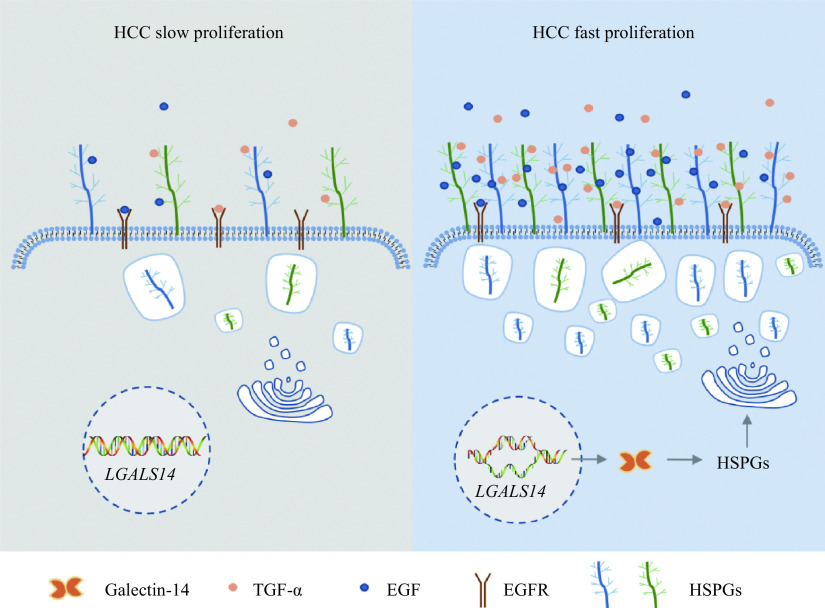
A schematic representation of how galectin-14 regulates hepatocellular carcinoma cells proliferation.

As the main component of ECM, HSPGs can recruit cytokines, chemokines, and growth factors, and thus protect them from proteolytic degradation, or work as co-receptors to regulate tumor cell proliferation by modulating the recognition of ligands and receptors ^[ 23, 30] ^. Because galectin-14 affected the expression of HSPGs in Huh-7 cells, we then investigated whether galectin-14 mediated the responsiveness of Huh-7 cells to growth factor stimulation. We treated Huh-7 cells with EGF and TGF-α to stimulate their proliferation. The results showed that wild-type Huh-7 cells showed a significant increase in proliferation in response to EGF and TGF-α treatment, while galectin-14 knockdown Huh-7 cells did not show a significant growth factor-induced proliferation ( ***Fig. 5A*** and ***5B***). We also performed RNA-seq analysis of the wild-type and galectin-14 knockdown Huh-7 cells, and found that the receptors for EGF and TGF-α were not significantly altered ( ***Fig. 5C***), indicating that those differences were caused by different sensitivity of Huh-7 cells responding to growth factor treatment but not the expression level of the receptor. Taken together, all our results indicate that galectin-14 promotes Huh-7 cells proliferation by increasing HSPG expression on cell surface to sensitize the cells to growth factors ( ***Fig. 6***).

## Discussion

In the current study, we identified a novel onco-fetal molecule galectin-14 in HCC by a single cell clone-based screening, and demonstrated that galectin-14 could increase the expression of HSPGs, causing tumor cells sensitize to growth factor and consequently promoting HCC tumor growth.

Intratumoral heterogeneity is a fundamental feature of many malignancies, including HCC, which limits the identification of novel therapeutic targets ^[ 31] ^. In recent years, the emergence of single-cell sequencing studies have revealed quite detailed intratumoral heterogeneity profiles ^[ 32] ^. However, it still can not obtain the omics profile and the function of the single cell at the same time ^[ 33– 35] ^. With these considerations, we obtained single Huh-7 cell clones, compared their tumor growth rate *in vitro* and *in vivo*, selected those clones with significantly different growth rates to perform RNA-seq analysis, and finally identified galectin-14 as a candidate molecule regulating HCC tumor growth. We found that galectin-14 expression gradually decreased in the single cell clones with the decreased growth rate, indicating that galectin-14 may represent the intratumor heterogeneity of HCC.

Previous studies have shown that galectins play important roles in HCC, *e.g.*, galectin-1 and galectin-3 were increased in HCC cells, compared with their normal counterparts, and the overexpression of galectin-1 and galectin-3 was significantly associated with a poor overall survival and might be considered as predictive prognostic factors ^[ 17– 18] ^. However, these proteins are also expressed in normal tissues, which limits their usefulness as specific biomarkers of HCC ^[ 36– 37] ^. The bio-distribution detection by a normal cDNA array showed that galectin-14 was expressed only in the placenta but not in normal tissues. Such ideal tumor specificity may make galectin-14 a good biomarker or therapeutic target. Theoretically, targeting galectin-14 in HCC would only have a minor off-tumor effect because galectin-14 was only expressed in adult placenta. Therefore, we could further design a new strategy to intervene in the expression of galectin-14 to attenuate HCC tumor growth. In this case, we could directly knock down the expression of galectin-14 by CRISPR-Cas9 or another strategy, or intervene in the regulation of galectin-14 expression. The latter mechanism of the aberrant expression of galectin-14 in HCC is still unclear, which needs further investigation.

Human galectin-14 is a novel prototype galectin in a dimer format ^[ 38] ^. Compared with other prototype galectins, each monomer of galectin-14 maintains the canonical "jelly-roll" fold, but the shape of the dimer is significantly different. Galectin-14 dimerizes not through its N and C termini or F-face, but *via* the interactions between its β-strands S5 and S6. Besides, galectin-14 has several natural mutation sites (Gln53 and His57) within the canonical ligand-binding site, which results in a much weaker ligand-binding activity than galectin-1, galectin-3, and galectin-8 ^[ 36, 38] ^. The unique structure features of galectin-14 may lead to its different functions from other galectins. Our results show that galectin-14 can affect glycoside synthesis, especially HSPGs, in Huh-7 cells. As we known that HSPGs are the key components of the ECM and function as the co-receptor of many growth factors and chemokines. In the current study, when we inhibited the expression of HSPGs in Huh-7 cells by knocking down EXT1, a rate-limiting enzyme in HSPG synthesis, the Huh-7 cell proliferation was significantly reduced. However, such an effect disappeared in galectin-14 knockdown Huh-7 cells. These observations indicate that galectin-14 may mediate the HSPGs-dependent tumor growth. Therefore, the underlying mechanism of how galectin-14 regulates the expression of HSPGs is quite necessary to be deeply explored in our future investigations.

Altogether, here we discover a novel HCC specific molecule that promotes HCC tumor growth by increasing the HSPGs-mediated growth factor response. Our findings reveal a novel mechanism of tumor progression and provide a potent target, which may be meaningful for developing new diagnostic and therapeutic strategies for HCC.
